# Peripheral polyneuropathy in severely obese patients with metabolic syndrome but without diabetes: association with high blood pressure, bmi and low HDL-cholesterol

**DOI:** 10.1186/1758-5996-7-S1-A112

**Published:** 2015-11-11

**Authors:** Otto Henrique Nienov, Luciana Matte, Lisiane Stefani Dias, Helena Schmid

**Affiliations:** 1 Universidade Federal de Ciências da Saúde de Porto Alegre, Porto Alegre, Brazil

## Background

Peripheral polyneuropathy (PPN) related to diabetes has been reported in association with causal factors such as obesity, hypertriglyceridemia, systemic arterial hypertension (SAH) and metabolic syndrome (MS), changes which frequently precede diabetes. To evaluate the prevalence of PPN in subjects with grade 2 and 3 obesity with MS without diabetes and to investigate for possible associating factors.

## Materials and methods

A cross-sectional study performed with grade 2 and 3 obese subjects with MS and without a diagnosis of diabetes using the Michigan Neuropathy Screening Instrument (MNSI) to assess the presence of PPN. Results: A total of 46 of 218 obese patients grade 2 and 3 with MS and without diabetes had PPN. From the variables studied, SAH (p=0.003), mean blood pressure (MBP) (p<0.001), low HDL-cholesterol (p=0.011), serum levels of HDL-cholesterol (p=0.048), BMI (p=0.036) and waist circumference (p=0.035) were significantly associated with PPN. There was a tendency for serum triglyceride levels (p=0.107) to associate with the presence of PPN. After multivariate regression, SAH, low HDL-cholesterol, BMI and waist circumference remained independently associated.

## Conclusion

Low levels of HDL-cholesterol, hypertension and increase of BMI and waist circumference are associated with PPN defined by the MNSI in patients with severe obesity and metabolic syndrome but without diabetes.

